# Supplementary material for: Improving Rice Leaf Shape Using CRISPR/Cas9-Mediated Genome Editing of SRL1 and Characterizing Its Regulatory Network Involved in Leaf Rolling through Transcriptome Analysis
Source: Int J Mol Sci. 2023 Jul 4;24(13):11087. doi: 10.3390/ijms241311087 (PMC10342453; doi:10.3390/ijms241311087)
Supplement: Supplementary file 1 [file ijms-24-11087-s001.zip › ijms-2481044-supplementary.pdf]

Table S1. Primer sequences used in this study

Name of primer	Primer sequences
SRL-F	TATGTGTGTGGCCACTGCTC
SRL-R	TTTGCTTTGCAGATGATGGG
gRT-SRL1	GCAGGTGTGCCCAATTCATTGTTTTAGAGCTAGAAAT
OsU6a-SRL1	AATGAATTGGGCACACCTGCCGGCAGCCAAGCCAGCA
gRT-SRL2	GGCGGTTTGACAAGCTACGCGTTTTAGAGCTAGAAAT
OsU6b-SRL1	GCGTAGCTTGTCAAACCGCCCAACACAAGCGGCAGC
U-F	CTCCGTTTTACCTGTGGAATCG
gR-R	CGGAGGAAAATTCCATCCAC
Pps-GGL	TTCAGAGGTCTCTCTCGACTAGTATGGAATCGGCAGCAAAGG
Pgs-GG2	AGCGTGGGTCTCGTCAGGGTCCATCCACTCCAAGCTC
Pps-GG2	TTCAGAGGTCTCTCTGACACTGGAATCGGCAGCAAAGG
Pgs-GGR	AGCGTGGGTCTCGACCGACGCGTATCCATCCACTCCAAGCTC
hyg-F	AAATCCGCGTGCACGAGGT
hyg-R	TCGTTATGTTTATCGGCACTTTGCA
Cas9-F	CTGACGCTAACCTCGACAAG
Cas9-R	CCGATCTAGTAACATAGATGACACC

Table S2. 459 differentially expressed genes detected by RNA-seq

MH63RS3	MSU	Type	log2 Fold Change	*p* Value
OsMH_01G0028800	*LOC_Os01g04040*	up	6.40620257	2.82×10^{-14}
OsMH_01G0037700	*LOC_Os01g04730*	up	1.380164442	0.000639305
OsMH_01G0061200	-	up	1.803551267	9.60×10^{-5}
OsMH_01G0079100	*LOC_Os01g09150*	up	2.860599778	0.000745064
OsMH_01G0140600	*LOC_Os01g15100*	up	3.925074433	7.18×10^{-5}
OsMH_01G0142800	-	up	2.609563837	0.000100655
OsMH_01G0152500	*LOC_Os01g16124*	up	2.812013889	0.000243742
OsMH_01G0153500	*LOC_Os01g16200*	up	2.885069341	2.43×10^{-5}
OsMH_01G0158100	*LOC_Os01g16600*	up	1.584752078	0.000709974
OsMH_01G0162900	*LOC_Os01g16960*	up	1.296802854	6.97×10^{-5}
OsMH_01G0239600	*LOC_Os01g24980*	up	2.084902352	0.000945955
OsMH_01G0251200	-	up	5.433755462	7.58×10^{-47}
OsMH_01G0253500	-	up	2.267089479	0.000385165
OsMH_01G0264300	-	up	3.062617952	7.33×10^{-5}
OsMH_01G0267300	*LOC_Os01g28450*	up	5.253063431	4.47×10^{-14}
OsMH_01G0299800	*LOC_Os01g31980*	up	2.403119579	6.58×10^{-5}
OsMH_01G0368100	*LOC_Os01g38650*	up	3.814982008	0.000205774
OsMH_01G0435700	*LOC_Os01g45640*	up	2.569400646	0.000185681
OsMH_01G0454200	*LOC_Os01g47580*	up	5.473634012	5.96×10^{-9}
OsMH_01G0467100	*LOC_Os01g48940*	up	2.399064569	4.33×10^{-5}
OsMH_01G0480800	-	up	4.954289111	3.69×10^{-9}
OsMH_01G0498200	*LOC_Os01g52240*	up	3.134023506	2.69×10^{-6}
OsMH_01G0505200	*LOC_Os01g52864*	up	1.189617825	0.00014148
OsMH_01G0538700	*LOC_Os01g56420*	up	1.544389348	1.79×10^{-5}
OsMH_01G0544000	*LOC_Os01g57004*	up	2.71762245	0.000980602
OsMH_01G0556000	*LOC_Os01g58290*	up	2.2874176	0.000119973
OsMH_01G0573800	*LOC_Os01g59950*	up	1.184145265	0.000786921
OsMH_01G0668200	*LOC_Os01g70240*	up	1.779462463	0.000687942
OsMH_01G0679800	*LOC_Os01g71474*	up	3.361982531	3.52×10^{-7}
OsMH_01G0679900	*LOC_Os01g71350*	up	3.325632392	0.00014919
OsMH_01G0681300	-	up	10.28947723	3.03×10^{-15}
OsMH_01G0690900	*LOC_Os01g72630*	up	7.757792241	2.42×10^{-39}
OsMH_02G0010100	-	up	2.019363488	8.10×10^{-7}
OsMH_02G0023200	*LOC_Os02g03360*	up	6.563109162	0.000164115
OsMH_02G0027200	*LOC_Os02g03710*	up	2.359001584	3.37×10^{-7}
OsMH_02G0032400	*LOC_Os02g04340*	up	7.318979892	2.80×10^{-6}
OsMH_02G0035700	*LOC_Os02g04690*	up	11.38392707	7.10×10^{-20}
OsMH_02G0038300	*LOC_Os02g05000*	up	12.51041649	1.23×10^{-23}
OsMH_02G0039400	-	up	8.933180957	6.85×10^{-9}
OsMH_02G0039500	-	up	8.512698354	6.23×10^{-10}
OsMH_02G0040600	*LOC_Os02g05190*	up	10.66816485	2.01×10^{-17}
OsMH_02G0040900	*LOC_Os02g05240*	up	7.023773064	4.55×10^{-5}

OsMH_02G0041000	*LOC_Os02g05244*	up	10.0851824	3.23×10^{-15}
OsMH_02G0041100	-	up	10.00064431	4.22×10^{-15}
OsMH_02G0041200	-	up	9.543288835	2.20×10^{-13}
OsMH_02G0041300	-	up	11.32556268	6.05×10^{-20}
OsMH_02G0043100	*LOC_Os02g05365*	up	10.35623992	2.10×10^{-12}
OsMH_02G0043800	*LOC_Os02g05430*	up	8.46673276	7.49×10^{-10}
OsMH_02G0044600	*LOC_Os02g05120*	up	6.570509814	0.000218722
OsMH_02G0047400	*LOC_Os02g05686*	up	11.06333786	5.25×10^{-17}
OsMH_02G0048800	*LOC_Os02g05810*	up	6.680132385	5.33×10^{-18}
OsMH_02G0051300	*LOC_Os02g06070*	up	3.001187833	8.12×10^{-5}
OsMH_02G0052700	*LOC_Os02g06205*	up	7.102705377	7.90×10^{-36}
OsMH_02G0056100	-	up	4.24758276	1.71×10^{-26}
OsMH_02G0057200	*LOC_Os02g06630*	up	2.796603071	0.000243431
OsMH_02G0080500	-	up	2.817407269	3.14×10^{-26}
OsMH_02G0085400	*LOC_Os02g09790*	up	1.909197284	6.53×10^{-6}
OsMH_02G0092500	*LOC_Os02g10500*	up	1.88014763	5.85×10^{-6}
OsMH_02G0093600	*LOC_Os02g10614*	up	7.810069599	3.96×10^{-7}
OsMH_02G0097000	-	up	1.37450596	0.000567954
OsMH_02G0102900	*LOC_Os02g11870*	up	2.792153847	0.000258365
OsMH_02G0105900	*LOC_Os02g12090*	up	1.675443933	6.13×10^{-5}
OsMH_02G0125300	*LOC_Os02g13870*	up	4.331573916	0.000196634
OsMH_02G0129100	-	up	6.229502417	8.83×10^{-9}
OsMH_02G0131700	*LOC_Os02g14440*	up	3.802818935	3.60×10^{-8}
OsMH_02G0142800	*LOC_Os02g15540*	up	1.763319359	0.000170171
OsMH_02G0182600	-	up	5.475329071	1.65×10^{-8}
OsMH_02G0186700	*LOC_Os02g20040*	up	2.261227971	0.000384436
OsMH_02G0188800	-	up	2.783295166	0.000149851
OsMH_02G0322400	*LOC_Os02g32814*	up	1.135857793	0.000163241
OsMH_02G0324100	*LOC_Os02g32980*	up	1.935651888	0.000237562
OsMH_02G0349400	*LOC_Os02g35329*	up	2.528378412	1.71×10^{-5}
OsMH_02G0378000	*LOC_Os02g38190*	up	1.424770246	4.84×10^{-5}
OsMH_02G0383000	*LOC_Os02g38840*	up	2.283533533	0.000301181
OsMH_02G0392100	*LOC_Os02g39710*	up	1.936765955	2.47×10^{-5}
OsMH_02G0404500	*LOC_Os02g41510*	up	3.288010412	7.73×10^{-5}
OsMH_02G0409600	*LOC_Os02g41954*	up	3.472330995	2.52×10^{-7}
OsMH_02G0416000	*LOC_Os02g42470*	up	1.802654055	0.000253422
OsMH_02G0419000	*LOC_Os02g42800*	up	4.711941911	6.63×10^{-13}
OsMH_02G0467500	*LOC_Os02g47090*	up	2.8843975	2.13×10^{-5}
OsMH_02G0480500	*LOC_Os02g48210*	up	2.176183125	0.000343709
OsMH_02G0487800	*LOC_Os02g48850*	up	6.631163647	0.000217375
OsMH_02G0505600	*LOC_Os02g50460*	up	2.102629801	0.000558362
OsMH_02G0520100	*LOC_Os02g51930*	up	3.804722537	1.24×10^{-8}
OsMH_02G0530000	*LOC_Os02g52990*	up	1.357138434	7.70×10^{-5}
OsMH_02G0534200	*LOC_Os02g53380*	up	5.787730057	4.09×10^{-7}

OsMH_02G0567900	*LOC_Os02g56880*	up	2.503885846	5.38×10^{-7}
OsMH_02G0573100	*LOC_Os02g57350*	up	3.138810349	2.55×10^{-8}
OsMH_03G0003500	-	up	2.357802953	4.33×10^{-5}
OsMH_03G0003600	*LOC_Os03g01320*	up	2.835957921	0.000131673
OsMH_03G0007100	*LOC_Os03g01720*	up	7.033926053	7.81×10^{-5}
OsMH_03G0021300	*LOC_Os03g03330*	up	8.661875375	3.25×10^{-8}
OsMH_03G0026300	*LOC_Os03g03730*	up	1.280771122	1.24×10^{-5}
OsMH_03G0047100	*LOC_Os03g05840*	up	1.652700746	0.000481838
OsMH_03G0060500	-	up	6.188759016	2.20×10^{-25}
OsMH_03G0069400	*LOC_Os03g08330*	up	3.002753118	4.75×10^{-6}
OsMH_03G0071100	*LOC_Os03g08490*	up	1.84740658	0.000490621
OsMH_03G0156500	*LOC_Os03g16600*	up	2.191244574	1.55×10^{-5}
OsMH_03G0163300	*LOC_Os03g17200*	up	3.644673763	2.56×10^{-10}
OsMH_03G0163400	-	up	5.726837589	3.43×10^{-8}
OsMH_03G0165200	*LOC_Os03g17410*	up	3.772833876	2.53×10^{-21}
OsMH_03G0177700	*LOC_Os03g18779*	up	3.701568717	1.76×10^{-5}
OsMH_03G0178400	*LOC_Os03g18850*	up	3.66975585	2.59×10^{-6}
OsMH_03G0180500	*LOC_Os03g19090*	up	2.553424637	2.92×10^{-7}
OsMH_03G0210300	*LOC_Os03g22210*	up	3.694827051	2.66×10^{-5}
OsMH_03G0210800	*LOC_Os03g22270*	up	1.707006902	3.91×10^{-5}
OsMH_03G0215100	*LOC_Os03g22680*	up	2.597572582	0.000805791
OsMH_03G0279800	*LOC_Os03g29770*	up	1.481270267	0.000226684
OsMH_03G0280500	*LOC_Os03g29850*	up	1.716819577	0.000636973
OsMH_03G0290900	*LOC_Os03g30950*	up	3.087146186	0.000252968
OsMH_03G0346900	-	up	2.849941585	3.26×10^{-5}
OsMH_03G0361700	-	up	8.819935883	7.14×10^{-5}
OsMH_03G0371900	*LOC_Os03g38790*	up	1.169985862	0.000156705
OsMH_03G0397400	-	up	4.150506147	9.53×10^{-6}
OsMH_03G0415900	*LOC_Os03g42350*	up	1.346152619	0.000687263
OsMH_03G0416600	*LOC_Os03g42380*	up	6.719904745	5.29×10^{-12}
OsMH_03G0416700	-	up	6.60274872	0.000271218
OsMH_03G0438100	*LOC_Os03g44710*	up	1.552227121	0.000520038
OsMH_03G0444600	*LOC_Os03g45344*	up	1.487557577	0.00079036
OsMH_03G0450400	*LOC_Os03g45960*	up	4.854667189	0.000202857
OsMH_03G0451300	*LOC_Os03g46060*	up	3.505326344	3.47×10^{-6}
OsMH_03G0451400	*LOC_Os03g46070*	up	3.513079906	7.92×10^{-10}
OsMH_03G0452400	*LOC_Os03g46200*	up	3.039399437	7.12×10^{-5}
OsMH_03G0462100	*LOC_Os03g47280*	up	7.244167889	3.69×10^{-5}
OsMH_03G0471400	*LOC_Os03g48060*	up	2.146754097	3.61×10^{-9}
OsMH_03G0492200	*LOC_Os03g50160*	up	2.590647951	0.000388607
OsMH_03G0501400	*LOC_Os03g51090*	up	1.305944804	4.37×10^{-6}
OsMH_03G0513200	*LOC_Os03g52390*	up	4.807296607	3.86×10^{-8}
OsMH_03G0536700	*LOC_Os03g55230*	up	3.600053507	2.44×10^{-5}
OsMH_03G0536800	-	up	3.156039782	0.000133783

OsMH_03G0560500	*LOC_Os03g57640*	up	5.584345817	0.000618079
OsMH_03G0564700	*LOC_Os03g58010*	up	2.527119952	0.000163129
OsMH_03G0598100	*LOC_Os03g61840*	up	1.53822548	0.00019622
OsMH_04G0009500	-	up	1.569768659	0.00038656
OsMH_04G0040500	*LOC_Os04g05650*	up	2.582005581	0.000347056
OsMH_04G0040600	-	up	2.649050652	0.000638879
OsMH_04G0040700	*LOC_Os04g05700*	up	6.571059165	0.000605684
OsMH_04G0079600	*LOC_Os04g10010*	up	3.459705205	0.000488194
OsMH_04G0087600	*LOC_Os04g10924*	up	2.229797769	0.000407511
OsMH_04G0087700	-	up	2.279833024	0.000311351
OsMH_04G0106300	-	up	3.646430031	3.68×10^{-9}
OsMH_04G0120200	-	up	8.738805705	6.19×10^{-9}
OsMH_04G0136900	*LOC_Os04g16450*	up	2.323304675	3.15×10^{-6}
OsMH_04G0141600	-	up	7.119434657	7.56×10^{-6}
OsMH_04G0144200	*LOC_Os04g17660*	up	1.677136916	0.000597683
OsMH_04G0176100	*LOC_Os04g19740*	up	1.160757194	0.000610534
OsMH_04G0237900	*LOC_Os04g25440*	up	1.675714459	5.18×10^{-5}
OsMH_04G0238900	-	up	8.715382037	6.96×10^{-10}
OsMH_04G0303200	*LOC_Os04g31790*	up	3.376687966	3.39×10^{-10}
OsMH_04G0310800	*LOC_Os04g32620*	up	2.447287749	4.25×10^{-5}
OsMH_04G0316900	*LOC_Os04g33240*	up	2.355472859	0.000181879
OsMH_04G0330800	*LOC_Os04g34540*	up	7.630100549	3.13×10^{-7}
OsMH_04G0340900	*LOC_Os04g35800*	up	1.006822005	0.000483062
OsMH_04G0341000	*LOC_Os04g35790*	up	2.17667669	1.81×10^{-12}
OsMH_04G0356700	*LOC_Os04g38490*	up	7.942189676	3.45×10^{-8}
OsMH_04G0369900	*LOC_Os04g39814*	up	2.279553945	1.60×10^{-5}
OsMH_04G0385600	*LOC_Os04g41680*	up	6.562832794	1.33×10^{-19}
OsMH_04G0385800	-	up	5.106321001	7.74×10^{-7}
OsMH_04G0390500	-	up	4.25191587	7.75×10^{-6}
OsMH_04G0400700	-	up	2.509024228	0.000359294
OsMH_04G0404400	*LOC_Os04g43680*	up	3.509968486	6.90×10^{-7}
OsMH_04G0419900	*LOC_Os04g45130*	up	2.650292537	0.000276053
OsMH_04G0424600	*LOC_Os04g45510*	up	7.059172983	4.37×10^{-11}
OsMH_04G0424700	*LOC_Os04g45520*	up	7.305853929	6.00×10^{-12}
OsMH_04G0439000	*LOC_Os04g47059*	up	5.01931627	1.99×10^{-6}
OsMH_04G0472300	*LOC_Os04g50120*	up	1.108846129	0.000663219
OsMH_04G0481000	*LOC_Os04g51450*	up	7.021122586	6.23×10^{-5}
OsMH_04G0488400	*LOC_Os04g52180*	up	1.655704649	7.20×10^{-6}
OsMH_04G0489200	*LOC_Os04g52260*	up	2.15159767	0.000244195
OsMH_04G0491500	*LOC_Os04g52504*	up	2.226357648	0.000186017
OsMH_04G0495000	*LOC_Os04g52790*	up	8.172213163	9.38×10^{-8}
OsMH_04G0505000	*LOC_Os04g53720*	up	5.2899201	1.15×10^{-5}
OsMH_04G0506400	*LOC_Os04g53930*	up	3.504609345	5.12×10^{-5}
OsMH_04G0513100	*LOC_Os04g54740*	up	2.80711507	3.01×10^{-5}

OsMH_04G0553800	*LOC_Os04g58860*	up	2.849155412	0.000534487
OsMH_05G0002800	*LOC_Os05g01330*	up	2.794597009	0.000143156
OsMH_05G0011300	*LOC_Os05g02200*	up	2.225904506	2.29×10^{-6}
OsMH_05G0013500	*LOC_Os05g02450*	up	2.793120303	1.99×10^{-5}
OsMH_05G0035600	*LOC_Os05g05030*	up	2.651491605	9.49×10^{-10}
OsMH_05G0035900	*LOC_Os05g05080*	up	4.733289718	0.000113451
OsMH_05G0072600	*LOC_Os05g08750*	up	1.557089241	0.000600007
OsMH_05G0088500	-	up	7.155275492	1.08×10^{-5}
OsMH_05G0107900	*LOC_Os05g12481*	up	1.616091977	0.000475189
OsMH_05G0129500	-	up	1.079005509	0.000901077
OsMH_05G0134600	*LOC_Os05g15770*	up	3.32424874	4.48×10^{-5}
OsMH_05G0224200	-	up	2.080220376	6.09×10^{-5}
OsMH_05G0253100	*LOC_Os05g28740*	up	2.619064583	0.000153044
OsMH_05G0289600	*LOC_Os05g32350*	up	2.020884429	0.000116295
OsMH_05G0301100	*LOC_Os05g33400*	up	5.190415496	0.000210939
OsMH_05G0318400	*LOC_Os05g35010*	up	2.387184252	0.000547329
OsMH_05G0327400	*LOC_Os05g35910*	up	1.665031361	0.000873174
OsMH_05G0351200	*LOC_Os05g38680*	up	2.434676942	7.14×10^{-12}
OsMH_05G0361100	*LOC_Os05g39720*	up	2.267088177	0.000694093
OsMH_05G0387800	*LOC_Os05g43040*	up	1.028820736	0.00079452
OsMH_05G0392100	-	up	5.164971877	5.19×10^{-5}
OsMH_05G0396300	*LOC_Os05g43970*	up	1.755073383	9.75×10^{-5}
OsMH_05G0401600	*LOC_Os05g44630*	up	7.71343483	3.09×10^{-7}
OsMH_05G0419400	*LOC_Os05g46510*	up	7.571164623	0.000102196
OsMH_05G0422000	*LOC_Os05g46830*	up	5.245028089	0.000145518
OsMH_05G0424500	*LOC_Os05g47700*	up	4.772038461	4.12×10^{-8}
OsMH_05G0428900	-	up	6.686690511	0.000210058
OsMH_05G0434400	*LOC_Os05g48680*	up	2.971155374	2.56×10^{-5}
OsMH_05G0447200	*LOC_Os05g49940*	up	2.3849452	0.000107844
OsMH_05G0449000	*LOC_Os05g50190*	up	2.988687297	8.24×10^{-6}
OsMH_06G0024300	*LOC_Os06g03830*	up	3.008798708	1.11×10^{-8}
OsMH_06G0073300	*LOC_Os06g08610*	up	6.312229208	0.000626668
OsMH_06G0090700	*LOC_Os06g10520*	up	2.124600589	0.000909348
OsMH_06G0104000	*LOC_Os06g11800*	up	3.367135412	3.25×10^{-8}
OsMH_06G0104100	-	up	3.47805607	1.23×10^{-5}
OsMH_06G0105100	*LOC_Os06g11900*	up	5.967775016	3.39×10^{-8}
OsMH_06G0187200	*LOC_Os06g19990*	up	1.319573025	0.000298399
OsMH_06G0199700	*LOC_Os06g21369*	up	7.906309005	6.48×10^{-7}
OsMH_06G0211200	*LOC_Os06g22290*	up	3.915534816	0.000441687
OsMH_06G0255100	*LOC_Os06g24920*	up	2.129765503	1.18×10^{-8}
OsMH_06G0315900	*LOC_Os06g34450*	up	1.691279003	0.0001063
OsMH_06G0360300	-	up	1.505606445	0.000162046
OsMH_06G0435800	*LOC_Os06g45890*	up	1.710364841	0.000826789
OsMH_06G0442600	-	up	1.822371196	0.000726761

OsMH_06G0449800	*LOC_Os06g47200*	up	1.927539365	7.83×10^{-5}
OsMH_06G0472600	*LOC_Os06g49660*	up	3.323607521	0.0003915
OsMH_07G0008900	*LOC_Os07g01904*	up	2.128801243	2.04×10^{-5}
OsMH_07G0009500	*LOC_Os07g01960*	up	9.704633289	4.09×10^{-10}
OsMH_07G0015200	*LOC_Os07g02510*	up	3.382691686	9.57×10^{-9}
OsMH_07G0021200	*LOC_Os07g03170*	up	2.716587839	2.40×10^{-6}
OsMH_07G0031700	*LOC_Os07g04350*	up	2.843136213	1.14×10^{-6}
OsMH_07G0041300	*LOC_Os07g05360*	up	4.088081692	0.000375096
OsMH_07G0041500	*LOC_Os07g05370*	up	2.222564312	0.000949696
OsMH_07G0045200	*LOC_Os07g05700*	up	2.561987636	2.00×10^{-9}
OsMH_07G0046000	*LOC_Os07g05840*	up	6.816103881	8.01×10^{-6}
OsMH_07G0049700	*LOC_Os07g06390*	up	1.095368138	0.000524427
OsMH_07G0057300	-	up	6.502135876	0.000731939
OsMH_07G0057400	*LOC_Os07g07030*	up	5.368616324	4.51×10^{-8}
OsMH_07G0079900	-	up	2.035847094	0.000884723
OsMH_07G0090200	*LOC_Os07g09970*	up	1.537407938	0.0009093
OsMH_07G0096800	-	up	6.39726224	0.000800194
OsMH_07G0100400	-	up	9.255288745	1.56×10^{-9}
OsMH_07G0119400	-	up	6.658549796	0.000541181
OsMH_07G0123100	-	up	5.593630547	9.31×10^{-7}
OsMH_07G0136800	*LOC_Os07g13830*	up	6.991704354	0.000229929
OsMH_07G0201000	*LOC_Os07g19210*	up	2.03699542	1.05×10^{-5}
OsMH_07G0300100	-	up	1.640594847	2.19×10^{-5}
OsMH_07G0312100	*LOC_Os07g31610*	up	6.652002576	0.000164944
OsMH_07G0344400	*LOC_Os07g35540*	up	1.945967173	0.000676741
OsMH_07G0392200	*LOC_Os07g40000*	up	3.62869225	7.18×10^{-11}
OsMH_07G0425400	*LOC_Os07g43310*	up	1.193384099	0.000134282
OsMH_07G0427800	*LOC_Os07g43560*	up	3.951923877	1.82×10^{-26}
OsMH_07G0428000	*LOC_Os07g43570*	up	13.37928567	1.43×10^{-93}
OsMH_07G0429800	*LOC_Os07g43770*	up	1.70837662	1.85×10^{-5}
OsMH_07G0462600	*LOC_Os07g47750*	up	2.407639531	0.000881505
OsMH_07G0467000	*LOC_Os07g48010*	up	2.123208538	2.52×10^{-5}
OsMH_08G0006800	*LOC_Os08g01670*	up	7.020593905	1.50×10^{-5}
OsMH_08G0066000	-	up	2.500573461	0.000269892
OsMH_08G0082100	*LOC_Os08g09380*	up	3.975031685	8.14×10^{-5}
OsMH_08G0095200	*LOC_Os08g10612*	up	7.189344604	0.000920151
OsMH_08G0129200	*LOC_Os08g13430*	up	4.512944619	2.52×10^{-11}
OsMH_08G0130800	-	up	1.996900068	0.000534534
OsMH_08G0188100	-	up	6.465143806	3.75×10^{-11}
OsMH_08G0203300	-	up	10.46153943	2.36×10^{-16}
OsMH_08G0270600	*LOC_Os08g28240*	up	9.487339129	1.20×10^{-9}
OsMH_08G0357900	*LOC_Os08g35490*	up	2.115591756	3.26×10^{-9}
OsMH_08G0379300	*LOC_Os08g37660*	up	6.638949949	0.00049294
OsMH_08G0382200	*LOC_Os08g37930*	up	5.442731246	3.14×10^{-10}

OsMH_08G0412800	-	up	2.423163533	0.000585312
OsMH_08G0413300	*LOC_Os08g40990*	up	4.628568865	0.000621814
OsMH_08G0415900	*LOC_Os08g41290*	up	4.252084008	6.91×10^{-6}
OsMH_08G0419100	*LOC_Os08g41610*	up	8.727270126	9.04×10^{-11}
OsMH_08G0419500	-	up	6.772071205	8.85×10^{-5}
OsMH_08G0419600	*LOC_Os08g41650*	up	5.52148263	3.97×10^{-7}
OsMH_08G0420500	*LOC_Os08g41720*	up	2.98343207	6.31×10^{-6}
OsMH_08G0421100	*LOC_Os08g41780*	up	2.905477744	2.43×10^{-5}
OsMH_08G0424200	*LOC_Os08g42290*	up	1.642762368	3.32×10^{-6}
OsMH_08G0424400	*LOC_Os08g42310*	up	12.18699544	3.44×10^{-23}
OsMH_08G0424600	*LOC_Os08g42320*	up	7.310209867	2.23×10^{-5}
OsMH_08G0428700	*LOC_Os08g42700*	up	8.158712207	8.86×10^{-5}
OsMH_08G0432500	*LOC_Os08g43020*	up	2.647434019	9.33×10^{-14}
OsMH_08G0433400	*LOC_Os08g43140*	up	6.420739954	0.000356932
OsMH_08G0433900	*LOC_Os08g43190*	up	1.077336546	1.52×10^{-5}
OsMH_08G0452200	*LOC_Os08g45200*	up	1.965851679	0.000386298
OsMH_08G0452500	*LOC_Os08g45210*	up	1.383021401	0.000940638
OsMH_09G0030400	*LOC_Os09g04050*	up	3.368824031	0.000406695
OsMH_09G0092200	*LOC_Os09g10054*	up	7.2518241	2.77×10^{-5}
OsMH_09G0099400	*LOC_Os09g10780*	up	5.768796505	0.00037699
OsMH_09G0110800	-	up	6.613185058	0.000177132
OsMH_09G0112900	*LOC_Os09g11480*	up	1.514445192	0.000110663
OsMH_09G0183500	-	up	4.878716316	0.000256603
OsMH_09G0213500	*LOC_Os09g20820*	up	1.490279081	0.000676475
OsMH_09G0236200	*LOC_Os09g23300*	up	2.062419747	3.49×10^{-6}
OsMH_09G0302100	*LOC_Os09g29690*	up	4.429176467	9.91×10^{-5}
OsMH_09G0302500	*LOC_Os09g29710*	up	8.046310937	0.000294433
OsMH_09G0308600	*LOC_Os09g30350*	up	2.017422333	4.36×10^{-8}
OsMH_09G0309400	*LOC_Os09g30414*	up	2.649244924	0.000173277
OsMH_09G0378300	-	up	6.472547084	0.000363899
OsMH_09G0379400	-	up	3.077710517	2.82×10^{-28}
OsMH_10G0013800	-	up	9.564883139	1.67×10^{-13}
OsMH_10G0018200	-	up	6.890066572	5.80×10^{-5}
OsMH_10G0073500	-	up	4.723610524	0.000194249
OsMH_10G0081100	-	up	5.20269133	5.05×10^{-5}
OsMH_10G0087700	-	up	7.055720206	8.91×10^{-5}
OsMH_10G0182100	*LOC_Os10g20450*	up	6.423531111	0.000436965
OsMH_10G0225900	*LOC_Os10g25290*	up	3.17093007	1.78×10^{-5}
OsMH_10G0240800	*LOC_Os10g26940*	up	8.533320802	0.000202663
OsMH_10G0244700	*LOC_Os10g27430*	up	3.417902925	0.000495648
OsMH_10G0246200	*LOC_Os10g28080*	up	2.793190008	0.000222432
OsMH_10G0249200	*LOC_Os10g28350*	up	2.445400227	1.36×10^{-7}
OsMH_10G0271900	-	up	3.141540309	5.86×10^{-7}
OsMH_10G0277200	*LOC_Os10g31780*	up	3.522688496	0.000905224

OsMH_10G0283300	*LOC_Os10g32600*	up	3.818814848	1.01×10^{-8}
OsMH_10G0289300	-	up	7.059068113	1.99×10^{-19}
OsMH_10G0294100	*LOC_Os10g33620*	up	1.660096655	0.000200497
OsMH_10G0307600	*LOC_Os10g34840*	up	2.766444667	4.27×10^{-7}
OsMH_10G0314700	*LOC_Os10g35460*	up	1.50915409	0.000349404
OsMH_10G0323600	*LOC_Os10g36580*	up	2.586253079	2.34×10^{-7}
OsMH_10G0334700	*LOC_Os10g38040*	up	2.069628142	0.00059945
OsMH_10G0340200	*LOC_Os10g38740*	up	1.668051718	0.000284931
OsMH_10G0343700	-	up	3.450860466	3.20×10^{-9}
OsMH_10G0350600	-	up	11.99431964	8.62×10^{-21}
OsMH_10G0359400	*LOC_Os10g40620*	up	4.480796198	0.000837565
OsMH_10G0361800	*LOC_Os10g40900*	up	2.194399009	0.000654642
OsMH_10G0362100	*LOC_Os10g40950*	up	1.781918813	0.000740748
OsMH_10G0371500	*LOC_Os10g41980*	up	2.704855704	3.82×10^{-6}
OsMH_10G0372100	-	up	2.398595654	0.000861287
OsMH_11G0005500	-	up	2.203378935	2.51×10^{-7}
OsMH_11G0010400	-	up	4.036295758	2.80×10^{-6}
OsMH_11G0040900	-	up	9.432296401	1.60×10^{-10}
OsMH_11G0069200	*LOC_Os11g07950*	up	2.059901553	0.000940806
OsMH_11G0069400	-	up	6.093059732	9.91×10^{-5}
OsMH_11G0069500	-	up	4.861108674	0.000162919
OsMH_11G0070800	*LOC_Os11g08070*	up	6.859478968	0.00010609
OsMH_11G0092300	-	up	12.6742858	2.78×10^{-24}
OsMH_11G0092600	-	up	13.09031152	2.99×10^{-24}
OsMH_11G0092700	-	up	5.038019337	4.51×10^{-10}
OsMH_11G0095000	-	up	13.27089079	3.82×10^{-26}
OsMH_11G0096900	-	up	10.73723987	1.39×10^{-16}
OsMH_11G0119400	*LOC_Os11g14140*	up	2.427625171	1.21×10^{-8}
OsMH_11G0127900	*LOC_Os11g15040*	up	3.201617071	6.16×10^{-7}
OsMH_11G0178900	-	up	3.857210139	4.05×10^{-6}
OsMH_11G0179000	-	up	3.619395098	6.30×10^{-5}
OsMH_11G0209600	*LOC_Os11g24510*	up	1.213595471	0.000675709
OsMH_11G0238800	*LOC_Os11g26950*	up	3.386387782	4.92×10^{-7}
OsMH_11G0249500	-	up	8.443180071	4.48×10^{-8}
OsMH_11G0257200	*LOC_Os11g28910*	up	2.253790854	0.000951713
OsMH_11G0266500	*LOC_Os11g29510*	up	3.032556629	6.81×10^{-5}
OsMH_11G0284300	*LOC_Os11g30910*	up	6.560993008	0.000860601
OsMH_11G0292400	*LOC_Os11g31690*	up	2.079480915	5.60×10^{-5}
OsMH_11G0310500	*LOC_Os11g33394*	up	2.658181983	6.08×10^{-9}
OsMH_11G0313500	*LOC_Os11g34080*	up	1.351702766	0.000525596
OsMH_11G0313600	-	up	1.482326934	8.84×10^{-5}
OsMH_11G0324200	*LOC_Os11g35310*	up	1.883879697	5.82×10^{-5}
OsMH_11G0346200	-	up	4.184565211	9.91×10^{-22}
OsMH_11G0366400	*LOC_Os11g40850*	up	2.098691672	0.00080484

OsMH_11G0379200	*LOC_Os11g41770*	up	7.432241498	0.000123302
OsMH_11G0395300	-	up	9.138540998	1.54×10^{-27}
OsMH_11G0399400	*LOC_Os11g44340*	up	5.145355388	0.000860764
OsMH_11G0401400	*LOC_Os11g44420*	up	2.319880577	1.73×10^{-5}
OsMH_11G0410200	*LOC_Os11g44990*	up	2.291255937	2.48×10^{-6}
OsMH_11G0411200	-	up	2.131412677	0.000433075
OsMH_11G0417400	*LOC_Os11g45060*	up	4.357124245	0.000235417
OsMH_11G0418200	-	up	4.035100547	0.000649716
OsMH_11G0420500	-	up	2.478427355	2.26×10^{-5}
OsMH_11G0422400	-	up	1.995534	4.19×10^{-5}
OsMH_11G0437400	*LOC_Os11g45740*	up	2.315794357	2.22×10^{-5}
OsMH_11G0449500	*LOC_Os11g47600*	up	5.460140431	0.000499833
OsMH_12G0001600	-	up	2.057027445	0.000183874
OsMH_12G0004900	-	up	1.964600409	5.79×10^{-5}
OsMH_12G0006100	-	up	2.260668816	2.02×10^{-5}
OsMH_12G0008500	-	up	6.958547823	0.000350106
OsMH_12G0016700	*LOC_Os12g02310*	up	1.689739384	0.000617929
OsMH_12G0022400	*LOC_Os12g03220*	up	4.042360484	9.18×10^{-5}
OsMH_12G0050900	*LOC_Os12g06190*	up	2.346990006	2.85×10^{-7}
OsMH_12G0060700	*LOC_Os12g07310*	up	3.168617334	2.74×10^{-7}
OsMH_12G0063700	*LOC_Os12g07580*	up	2.42132824	1.22×10^{-8}
OsMH_12G0086100	*LOC_Os12g10750*	up	6.877214213	0.000254957
OsMH_12G0107200	-	up	7.358361264	5.33×10^{-6}
OsMH_12G0210900	*LOC_Os12g25630*	up	1.184941139	0.000977967
OsMH_12G0215100	*LOC_Os12g26290*	up	3.685772238	1.09×10^{-7}
OsMH_12G0236500	-	up	2.244042197	8.16×10^{-6}
OsMH_12G0259100	*LOC_Os12g31860*	up	6.74492892	0.00077124
OsMH_12G0262300	*LOC_Os12g32390*	up	3.755510356	0.000134439
OsMH_12G0297600	*LOC_Os12g36830*	up	2.704463032	9.73×10^{-5}
OsMH_12G0297800	*LOC_Os12g36850*	up	2.620840548	7.11×10^{-5}
OsMH_12G0311200	*LOC_Os12g38250*	up	3.114888006	0.000127069
OsMH_12G0322200	*LOC_Os12g39360*	up	2.748121916	0.000946565
OsMH_12G0360800	*LOC_Os12g43430*	up	3.756334305	6.84×10^{-5}
OsMH_12G0360900	*LOC_Os12g43390*	up	5.550807592	5.95×10^{-6}
OsMH_12G0361600	-	up	4.450799028	5.12×10^{-7}
OsMH_12G0361700	*LOC_Os12g43440*	up	5.08850721	0.000891035
OsMH_12G0361800	*LOC_Os12g43450*	up	8.298264469	5.51×10^{-7}
OsMH_12G0362000	-	up	4.289151625	0.000263878
OsMH_01G0017200	*LOC_Os01g02884*	down	-1.228625927	0.000251914
OsMH_01G0056900	*LOC_Os01g06790*	down	-1.760457228	2.37×10^{-7}
OsMH_01G0243700	*LOC_Os01g25484*	down	-1.574305962	0.00077853
OsMH_01G0383100	-	down	-6.524553912	0.000663148
OsMH_02G0008900	*LOC_Os02g01940*	down	-1.553560907	1.20×10^{-6}
OsMH_02G0009200	*LOC_Os02g01970*	down	-1.396179752	0.000425155

OsMH_02G0033000	*LOC_Os02g04410*	down	-9.01809689	1.75×10^{-11}
OsMH_02G0035600	*LOC_Os02g04680*	down	-1.904739599	2.11×10^{-9}
OsMH_02G0036700	*LOC_Os02g04780*	down	-1.464810654	3.59×10^{-6}
OsMH_02G0045600	*LOC_Os02g05530*	down	-11.43021943	2.52×10^{-19}
OsMH_02G0046300	*LOC_Os02g05600*	down	-2.541726566	1.05×10^{-6}
OsMH_02G0053000	-	down	-1.495929148	5.84×10^{-5}
OsMH_02G0060000	-	down	-6.877190579	1.58×10^{-10}
OsMH_02G0070200	*LOC_Os02g07830*	down	-1.184288827	0.000632693
OsMH_02G0081700	*LOC_Os02g09340*	down	-3.002155071	4.07×10^{-8}
OsMH_02G0478000	*LOC_Os02g47920*	down	-3.138281428	5.77×10^{-6}
OsMH_02G0494100	*LOC_Os02g49510*	down	-1.573968746	0.000633364
OsMH_02G0568900	*LOC_Os02g56940*	down	-2.122781968	0.00030371
OsMH_02G0569000	*LOC_Os02g56950*	down	-1.818671394	3.02×10^{-5}
OsMH_03G0129800	-	down	-2.311737036	9.86×10^{-5}
OsMH_03G0269400	*LOC_Os03g28300*	down	-1.375653528	0.000123129
OsMH_03G0292900	*LOC_Os03g31180*	down	-1.216606548	0.000923536
OsMH_03G0298100	-	down	-5.562862978	0.000115811
OsMH_03G0345300	-	down	-6.808545406	2.74×10^{-21}
OsMH_03G0356300	*LOC_Os03g37470*	down	-1.157865276	4.62×10^{-5}
OsMH_03G0371000	*LOC_Os03g38745*	down	-1.441468163	0.000545306
OsMH_03G0621900	*LOC_Os03g64415*	down	-7.107965206	1.35×10^{-5}
OsMH_04G0024100	-	down	-2.063320237	6.20×10^{-5}
OsMH_04G0328600	*LOC_Os04g34290*	down	-1.043392756	0.000246155
OsMH_04G0451800	*LOC_Os04g48410*	down	-1.241728248	0.000282453
OsMH_04G0559000	*LOC_Os04g59540*	down	-1.319248649	0.000838264
OsMH_05G0178200	*LOC_Os05g20450*	down	-2.101279578	8.34×10^{-6}
OsMH_05G0216900	*LOC_Os05g25350*	down	-1.560253293	0.000140394
OsMH_05G0303100	*LOC_Os05g33554*	down	-6.347901754	0.000657925
OsMH_05G0374800	*LOC_Os05g41270*	down	-6.468626131	0.000857969
OsMH_05G0406000	*LOC_Os05g45240*	down	-1.833387903	1.42×10^{-5}
OsMH_05G0465100	-	down	-10.01355152	1.95×10^{-14}
OsMH_06G0088200	*LOC_Os06g10280*	down	-1.116350656	0.000351812
OsMH_06G0378500	*LOC_Os06g40180*	down	-2.446131532	0.000228938
OsMH_07G0002300	*LOC_Os07g01240*	down	-1.502129676	2.41×10^{-5}
OsMH_07G0027800	*LOC_Os07g03870*	down	-2.416031547	9.55×10^{-5}
OsMH_07G0482100	*LOC_Os07g49460*	down	-1.073594976	0.000639235
OsMH_08G0091500	-	down	-9.115908839	2.31×10^{-10}
OsMH_08G0188800	*LOC_Os08g20590*	down	-6.706333307	0.000123785
OsMH_08G0413900	*LOC_Os08g41080*	down	-1.866552254	0.000169602
OsMH_08G0415700	*LOC_Os08g41270*	down	-1.249108697	2.09×10^{-5}
OsMH_08G0421800	-	down	-2.45979832	0.000181097
OsMH_08G0424000	*LOC_Os08g42268*	down	-2.924946193	7.30×10^{-6}
OsMH_08G0425900	-	down	-1.793197883	3.46×10^{-7}
OsMH_08G0426000	-	down	-2.430338917	1.41×10^{-13}

OsMH_08G0435100	-	down	-7.844324731	3.24×10^{-7}
OsMH_08G0435200	-	down	-8.838372523	8.47×10^{-11}
OsMH_08G0448400	*LOC_Os08g44840*	down	-1.192836988	0.000195723
OsMH_09G0187300	-	down	-1.290733586	0.000718554
OsMH_09G0287400	*LOC_Os09g28340*	down	-1.596898396	0.000904615
OsMH_09G0298700	*LOC_Os09g29390*	down	-1.997015175	0.000460872
OsMH_09G0374600	-	down	-1.490756414	3.94×10^{-5}
OsMH_09G0380200	*LOC_Os09g39020*	down	-4.051614166	1.76×10^{-5}
OsMH_09G0382000	*LOC_Os09g39320*	down	-1.622772438	6.50×10^{-6}
OsMH_10G0314200	-	down	-3.837627044	0.0001326
OsMH_11G0001700	*LOC_Os11g01154*	down	-5.215245815	7.39×10^{-6}
OsMH_11G0002100	-	down	-1.231927306	0.000481547
OsMH_11G0003500	*LOC_Os11g01330*	down	-1.568391152	0.00026424
OsMH_11G0107500	-	down	-1.539425108	1.21×10^{-5}
OsMH_11G0186200	-	down	-1.188280095	0.000775643
OsMH_11G0192600	*LOC_Os11g22350*	down	-1.50433013	0.000264031
OsMH_11G0262900	*LOC_Os11g29720*	down	-2.115045477	2.17×10^{-8}
OsMH_11G0283500	-	down	-2.126080642	0.000197386
OsMH_11G0345400	-	down	-6.830336374	0.000192886
OsMH_11G0409400	*LOC_Os11g44890*	down	-1.317589656	0.000688405
OsMH_12G0194100	*LOC_Os12g24550*	down	-1.690758575	0.000416069

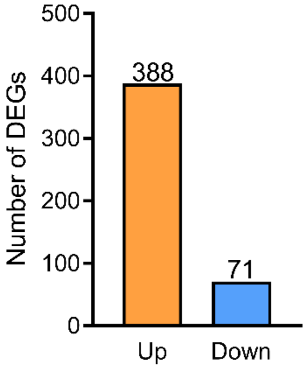

Figure S1. RNA-seq detected 459 differentially expressed genes, of which 388 genes were significantly upregulated, 71 genes were downregulated, fold change > 2, FDR < 0.05.

Table S3. Expression changes of *srl1-103* resistance-related genes

MSU	MH63RS3	Type	Description	log2 Fold Change	*p* Value
LOC_Os04g41680	*OsMH_04G 0385600*	up	CHIT3-chitinase family protein precursor	6.562832794	1.33×10^{-19}
LOC_Os02g14440	*OsMH_02G 0131700*	up	peroxidase precursor	3.802818935	3.60×10^{-8}
LOC_Os07g48010	*OsMH_07G 0467000*	up	peroxidase precursor	2.123208538	2.52×10^{-5}
LOC_Os04g43680	*OsMH_04G 0404400*	up	MYB family transcription factor	3.509968486	6.90×10^{-7}
LOC_Os11g45740	*OsMH_11G 0437400*	up	MYB family transcription factor	2.315794357	2.22×10^{-5}
LOC_Os01g28450	*OsMH_01G 0267300*	up	SCP-like extracellular protein	5.253063431	4.47×10^{-14}
LOC_Os03g18850	*OsMH_03G 0178400*	up	pathogenesis-related Bet v I family protein	3.66975585	2.59×10^{-6}
LOC_Os12g36830	*OsMH_12G 0297600*	up	pathogenesis-related Bet v I family protein	2.704463032	9.73×10^{-5}
